# Brain-gut communication and potential applications of microecological treatments in stroke

**DOI:** 10.3389/fnins.2026.1799890

**Published:** 2026-04-15

**Authors:** Mingzhu Gao, Yujie Zhou, Kaixin Zhang, Yang Ye, Zhijun Han, Qi Chen, Wentao Shi, Xiaojie Lu

**Affiliations:** 1 Department of Clinical Research Center, Jiangnan University Medical Center (Wuxi No.2 People’s Hospital), Wuxi School of Medicine, Jiangnan University, Wuxi, China; 2 Research Institute for Reproductive Health and Genetic Diseases, Wuxi Maternity and Child Health Care Hospital, Wuxi School of Medicine, Jiangnan University, Wuxi, China; 3 Department of Neurosurgery, Jiangnan University Medical Center (Wuxi No.2 People’s Hospital), Wuxi School of Medicine, Jiangnan University, Wuxi, China

**Keywords:** clinical applications, gut-brain axis, microecological agents, regulatory pathways, stroke

## Abstract

Stroke is a cerebrovascular disease with high incidence rates, serious disability and increased mortality rates, thereby posing a serious threat to human health. The mechanisms of brain-gut communication have gradually emerged in recent times. This article focuses on the gut-brain axis and discusses the bidirectional regulatory pathways between gut microecology and stroke via the neurotransmitter, colony metabolite, endocrine, and immunoregulatory pathways. Additionally, it summarizes the latest applications of gut microecological agents in stroke, which may provide new research ideas and clinical treatment strategies for the microecological diagnosis and therapy of stroke.

## 1 Introduction

As one of the leading causes of mortality and lifelong disability globally, stroke imposes significant economic, social and clinical burdens on patients, families and health services (GBD 2019 Stroke Collaborators, 2021; Kim et al., 2020). Particularly in low and middle income countries, stroke incidence continues to increase as a result of progressive global population aging and rise in the prevalence of cardiovascular risk factors, namely obesity, dyslipidemia, hypertension and diabetes mellitus (O'Donnell et al., 2016; Vaduganathan et al., 2022). The functional recovery post-stroke is often incomplete since there are limited effective treatment options for the condition despite advances in acute reperfusion therapies, such as mechanical thrombectomy and thrombolysis (Campbell and Khatri, 2020). To improve prevention, treatment and prognosis of stroke, identification of novel pathophysiological mechanisms and pharmacological targets are increasing demanded.

The role of gut microbiota in causing neurological diseases has gained increased attention in recent times. Gut microbiota collectively refers to a complex and dynamic microbial system, which is harbored in the human and is composed of archaea, fungi, viruses and bacteria (Sender et al., 2016). In view of their huge genetic repertoire, these microorganisms have been found to participate in physiological processes, such as maintenance of intestinal barrier integrity, maturation of immune system, protection against pathogenic invasion, synthesis of vitamins, and metabolism of nutrients (Sommer and Bäckhed, 2013; Round and Mazmanian, 2009). Various diseases such as cardiovascular diseases (CVDs), central nervous system (CNS) disorders, immune-mediated conditions and metabolic disorders developed as a result of dysbiosis (imbalance of gut microbiome) (Lynch and Pedersen, 2016; Tilg et al., 2020).

The gastrointestinal tract (GIT) is linked to the CNS by a two-way directional communication network known as gut-brain axis (GBA) via immune, metabolic and neural pathways (Cryan and Dinan, 2012). The development, function and behavior of the brain can be influenced by gut microbiota through this axis with the brain modulating the mobility, secretion, permeability and immune responses of the gut (Carabotti et al., 2015). Growing evidence suggests that the potential for gut microbiota-derived hormones, inflammatory mediators, neurotransmitters and metabolites to cross the barrier of intestines and occasionally the blood–brain barrier (BBB) can directly or indirectly affect neuroinflammatory processes and neuronal activity (Sharon et al., 2016; Fung et al., 2017). Acute brain injuries including stroke, neuropsychiatric conditions and neurogenerative disorders have been linked with GBA dysregulation (Sampson and Mazmanian, 2015; Dinan and Cryan, 2020; Góralczyk-Bińkowska et al., 2022).

Through emerging studies, scientists have discovered stroke as a systemic disease and focal cerebrovascular event that significantly impact function of gut and composition of microbiome (Chidambaram et al., 2022; Szegedi et al., 2025). Also, secondary brain function, systemic inflammation and immune dysfunction were exacerbated by acute cerebral ischemia or hemorrhage-induced intestinal dysmotility, increased permeability of gut and microbiota dysbiosis (Singh et al., 2016; Winek et al., 2016). In contrast, susceptibility and recovery of stroke, infarct size, and inflammation after stroke are influenced by pre-existing composition of gut microbiota and their metabolites (Benakis et al., 2016; Yamashiro et al., 2017). Based on available evidence, gut microbiota can be considered as a contributor to pathogenesis of stroke and as a potential target for treatment of the condition.

In this context, scientists have given much attention to microecological agents (MEAs), namely microbiota-derived metabolites, synbiotics, prebiotics and probiotics, which have been regarded as adjunctive schemes for prevention, treatment and rehabilitation of stroke (Zhong et al., 2021; Li et al., 2023). It is evidenced that the aforementioned interventions may regulate metabolic pathways, reduce neuroinflammation, ameliorate homeostasis of immune system and promote post-stroke recovery of neurons by modulating of composition and function of gut microbiome (Sadler et al., 2020). Nevertheless, scientists have not clearly understood the mechanisms that underlie interaction of microbiota–brain in stroke, while further systematic evaluation of clinical translation of microbiome-based therapies is required.

**Figure 1:**
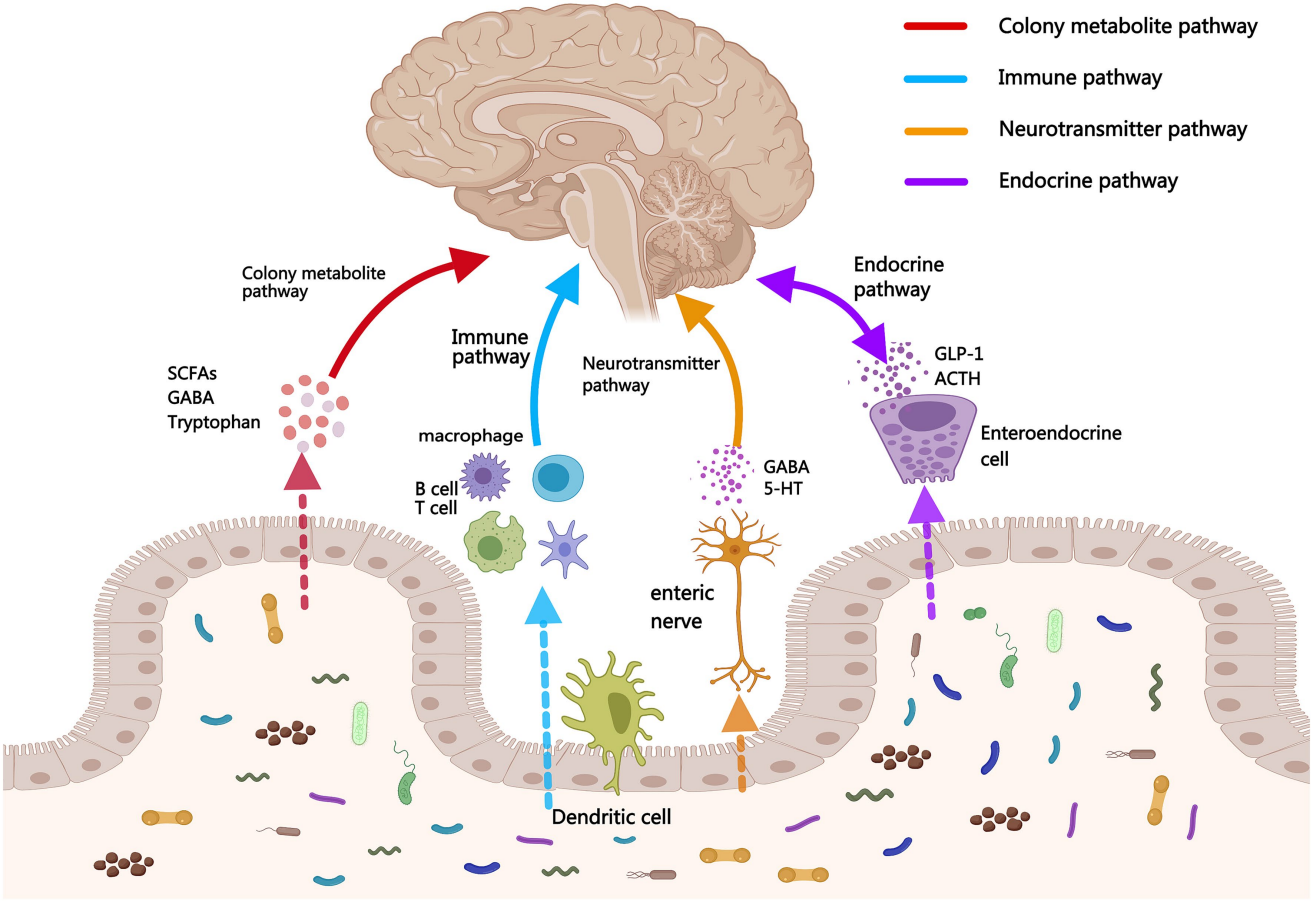
Four major regulatory pathways of brain-gut dialogues.

This article explores four major regulatory pathways and mechanisms involved in brain-gut communication based on the concept of the GBA (Figure 1). It also summarizes the cutting-edge applications of MEAs in the diagnosis and treatment of stroke (Table 1), thus providing new research ideas and clinical treatment approaches for microecological diagnosis and stroke treatment.

**Table 1:** Cutting-edge applications of microecological agents in stroke.

Microecological preparation	Clinical applications	Beneficiaries
Probiotics combined with enteral nutrition (Petrella et al., 2021)	Probiotics Combined with Enteral Nutrition Improves Nutritional Status and Reduces TNF-α, IL-6, and IL-10 Levels	Ischemic stroke after thrombolysis
*Bifidobacterium bifidum* Tetragonum Tablets (Fu et al., 2023)	Oral *Bifidobacterium tetragonum* tablets improve nutritional status, gut microbiota and intestinal mucosal barrier function of the body	Postoperative severe hemorrhagic stroke
Early enteral nutrition combined with synbiotics	Early enteral nutrition with added synbiotics improves the imbalance of gut microbiota and upregulates the local immune function of the intestines.	Hypertensive hemorrhagic stroke
*Bifidobacterium bifidum* preparation (Ren et al., 2023)	Oral bifidobacteria improve cognitive function and regulate lipid and glucose metabolism in IS patients	Post-stroke cognitive dysfunction
Mixed preparations of *Lactobacillus acidophilus*, *Lactobacillus casei* and *Lactobacillus fermentum* (Menke, 2019)	Oral probiotic blend improves patients’ cognitive status and insulin metabolism	Post-stroke cognitive dysfunction
Short-chain fatty acid blend (Xie et al., 2024)	SCFA supplementation improves recovery, cortical remodeling, and synaptic plasticity after stroke	Post-stroke depression
*Bifidobacterium longum*, *Lactobacillus rhamnosus* and other mixed probiotics (Wang et al., 2022)	Improvement in Depression and Anxiety Symptoms After Oral Probiotic Blend	Post-stroke depression
Enteral Nutrition with *Bifidobacterium* Triplex Capsules (Chen X. et al., 2022)	Enteral nutrition combined with *Bifidobacterium breve* triplex capsules improves neurological dysfunction and immune function in patients	Post-stroke dysphagia

## 2 Gut microecology and GBA

Gut micro-ecosystem denotes the complex microbial community consisting of various microorganisms that reside in the gut (Yang et al., 2023). The genetic diversity of these microorganisms ensure that they capably perform several functions. These principal roles include assisting fermentation and digestion of food, generating some vitamins, promoting absorption of nutrients, facilitating development of gut-associated lymphoid tissues, and preventing colonization of harmful foreign microorganisms (Xu et al., 2021). Countless bacteria interdependently and mutually restrictively coexist and grow within the gut. There is disruption of micro-ecosystem balance (also known as dysbiosis), thereby culminating in corresponding symptoms in the intestines and even triggering systemic alterations (Abdel-Haq et al., 2019).

The brain’s cognitive and emotional centers with intestinal function are connected by GBA, which acts as a two-way directional information exchange pathway between intestinal nervous system and CNS (Agirman et al., 2021). A growing number of studies have disclosed significance of GBA in health disease in recent times as research on this axis deepens. The involvement of gut microbiota in the interaction of intestines with nervous system aids the maintenance of gastrointestinal homeostasis and impacts increased cognitive brain functions (Schneider et al., 2024). As stated in the preceding section, brain behavior is influenced when some microbiota, related inflammatory mediators, neuroactive and molecular active metabolites cross the BBB and intestinal barriers, thereby propagating along the brain-gut-microbiota system (Lof et al., 2022). The brain function and host behavior can be significantly impacted by involvement of gut microbiota in the synthesis and release of active metabolites, immune responses, several hormones and neurotransmitters (Aburto and Cryan, 2024).

## 3 Four major regulatory pathways of gut-brain communication in stroke

Stroke is a group of diseases caused by sudden rupture of blood vessels in the brain or blockage of blood flow, which leads to damage to brain tissue (Feigin et al., 2025). Hemorrhage stroke and ischemic stroke (IS) are the two principal categories of strokes based on the cause. Of the two categories of strokes, IS is considered the most common type, accounting for 70–80% of all stroke cases (Shekarabi et al., 2024). Recent study has demonstrated that gut microbiota could substantially influence onset and progression of stroke (Peh et al., 2022). Through direct or indirect mechanisms, gut microbiota impacts the metabolism of the host, keeps mucosal barrier function of intestines, and regulates function of the brain (Fan et al., 2023). In particular, various pathways such as immune responses, hormonal factors, microbial metabolites and neurotransmitters mediate bidirectional communication between gut microbiota and CNS, which overall form the basis of communication within GBA. Importantly, these mechanisms are not activated in a static microbial background, because stroke itself can rapidly alter gut microbial composition and function, thereby reshaping the downstream signaling pathways of the gut-brain axis. It is postulated that regulation of gut microbiota may effectively attenuate stroke, reduce complications after stroke and promote recovery of patients. Notwithstanding, there is urgent need for detailed research into these regulatory mechanisms to improve the prevention, treatment and prognosis of stroke. This may aid understanding of the principles that underlie GBA and provide foundational basis for development of novel microbiome-based treatment strategies (Fan et al., 2023).

### 3.1 Stroke-associated gut microbiota dysbiosis as an upstream driver of the four regulatory pathways

Before discussing the four major regulatory pathways individually, it is important to recognize that stroke itself can induce profound alterations in gut microbiota composition (Peh et al., 2022). Both ischemic and hemorrhagic stroke have been associated with reduced microbial diversity, depletion of beneficial commensals, enrichment of opportunistic pathogens, and a decline in short-chain fatty acid (SCFA)-producing bacteria (Zhang W. et al., 2024; Haak et al., 2021). These changes are often accompanied by impaired intestinal motility, increased gut permeability, and disruption of mucosal homeostasis (Shen et al., 2025). Therefore, stroke-associated dysbiosis should not be viewed merely as a secondary phenomenon, but rather as an important upstream event that may reshape gut-brain communication in the context of stroke (Hendel et al., 2026).

Such microbial alterations may influence all four regulatory pathways discussed below. First, dysbiosis may alter the synthesis, availability, and signaling effects of neuroactive molecules, thereby affecting neurotransmitter-related communication between the gut and the brain (Pluta et al., 2021). Second, depletion of beneficial anaerobes and SCFA-producing bacteria may disturb production of key microbial metabolites involved in neuroprotection, barrier maintenance, and metabolic homeostasis (Zhang W. et al., 2023a). Third, together with increased intestinal permeability, stroke-induced dysbiosis may reinforce neuroendocrine stress signaling by sustaining hypothalamic–pituitary–adrenal axis activation and disturbing gut endocrine function (Benakis and Liesz, 2022). Finally, enrichment of pathogenic bacteria and increased translocation of microbial products such as lipopolysaccharide (LPS) may amplify peripheral and central immune activation (Wang T. et al., 2023). In this way, stroke-associated changes in gut microbiota composition provide a mechanistic bridge linking the initial cerebrovascular insult to subsequent dysregulation of neurotransmitter, metabolic, endocrine, and immune pathways along the GBA (Zhang S. et al., 2023). Against this background, we discuss how stroke-associated dysbiosis may affect the neurotransmitter, microbial metabolite, endocrine, and immunomodulatory pathways in a more pathway-specific manner in the following sections.

### 3.2 Neurotransmitter pathways

Two chief neural connections exist between the brain and gut. In the first neural connection, there is communication through the autonomic nervous system within spinal cord and vagus nerve. In the second neural connection, the intestinal nervous system is exploited for bidirectional information exchange (de Weerth, 2017). The gut and brain capably influence each other through these pathways. Bacteria stimulate the gut neurons and vagus nerve to institute direct neural connections between the brain and gut microbiota. The vagus nerve regulates gut function through the excitatory cholinergic reflexes, thereby playing a vital role during injury to gut mucosa (Moradian et al., 2024). Several neurotransmitters and their precursors that are produced by the gut microbiota (Table 2) impact neuronal brain activity. For instance, *Bacillus cereus* can synthesize norepinephrine and glutamate in the gut, while *Escherichia coli* can generate norepinephrine (Morais et al., 2021). Existing report suggests that norepinephrine can stimulate migration of bacteria toward the intestinal mucosa of the host and increase expression of pathogenic genes through its interaction with bacterial sensory mechanisms (Lopes and Sourjik, 2018). After a stroke, the risk of infection increases due to gut microbiota imbalance and concomitant higher levels of norepinephrine (Wang Q. et al., 2024). In the CNS, one of the most common excitatory neurotransmitters is glutamate (Thoreson and Witkovsky, 1999). The astrocytes and neurons release elevated glutamate levels under ischemic conditions, which suggests that it is a significant factor for degeneration of neurons (Jiang et al., 2021). Glutamate accumulation in the extracellular environment is mediated by certain factors, namely mechanisms of impaired reuptake, dysfunction of ion channel and disorders of energy metabolism, thereby increasing sensitivity of the brain to ischemic injury. During the process of neuronal death, there is monumental influx of sodium ions, calcium ions and water molecules into neurons due to excessive glutamate levels, which continuously activate receptors of N-methyl-D-aspartate (Freire-Cobo et al., 2014). Studies have shown that elevated glutamate levels in patients’ blood and cerebrospinal fluid positively correlated with worsening neurological function (Yang et al., 2019). Additionally, high glutamate levels have been closely associated with occurrence of acute lung injury, an important complication of stroke, thereby predicting poor outcomes. As the primary inhibitory neurotransmitter in nervous system, gamma-aminobutyric acid (GABA) can be produced from glutamate by both human body and gut microbiota (Xu et al., 2023). Some species of *Bifidobacterium* and *Lactobacillus* can utilize glutamate to produce GABA through the glutamine-glutamate/GABA metabolic cycle (Samara et al., 2022). In cases of glutamate release due to ischemic injury, GABA may be released simultaneously, counteracting neuronal overexcitability by hyperpolarizing neuronal membrane potential and blocking glutamate excitatory transmission (Strandwitz et al., 2019). Also, 5-Hydroxytryptamine (5-HT) is an important inhibitory neurotransmitter. *Clostridium difficile* in the gut can promote serotonin synthesis and produce specific metabolites by increasing gene expression of tryptophan hydroxylase-1 (a rate-limiting enzyme) in colonic chromaffin cells (Wang Z. J. et al., 2023). Dysregulated gut microbiota may disrupt peripheral 5-HT levels, affecting its interaction with neuronal receptors in the enteric nervous system, thereby regulating intestinal motility (de Paiva et al., 2024). This disruption may also impact neuronal development and differentiation processes. Through the vagus nerve, gut microbiota can influence the nucleus tractus solitarius and dorsal raphe nucleus, thereby regulating mood (Kesika et al., 2021). In summary, the gut microbiota influences occurrence and development of stroke and its complications by influencing neurotransmitters synthesis, namely 5-HT and norepinephrine. Studies have also shown that GABA levels below 240 nmol/L can serve as a positive predictive marker for post-stroke neurological deterioration (He et al., 2019).

**Table 2:** Gut microbiota enables brain-gut dialogue via four major pathways.

Pathways of influence	Target of action	Associated microbiota	Function overview
Neurotransmitter pathways	Norepinephrine	*Escherichia coli* and *Bacillus*	Elevated norepinephrine levels can promote bacterial migration to the intestinal mucosa and enhance the expression of virulence genes (Parker et al., 2020).
	γ-Aminobutyric acid	*Lactobacillus* and *Bifidobacterium*	Gamma-aminobutyric acid (GABA) counteracts excitotoxicity in neurons by hyperpolarizing the membrane and inhibiting glutamatergic transmission (Bai et al., 2022).
	5-Hydroxytryptophan	*Clostridium difficile*	Promotes gene expression of tryptophan hydroxylase 1 in enterochromaffin cells and enhances serotonin biosynthesis (Chidambaram et al., 2022).
Colony metabolite pathways	Short-chain fatty acid	Anaerobic bacteria, *Clostridium difficile*	Short-chain fatty acids (SCFAs) exert anti-inflammatory and neuroprotective effects by inhibiting NF-κB activation and promoting Erk1/2 activation (Kumar et al., 2021).
	Bile acids	*Bifidobacteria* and *bacilli*	Through the activation of FXR signaling, bile acids can induce the host to express antimicrobial defense genes, thereby reducing intestinal epithelial damage and bacterial translocation (Bender et al., 2023).
	Trimethylamine oxide	Anaerobic cocci, *Clostridium perfringens*	TMAO can promote thrombosis in atherosclerosis by increasing the expression and activity of tissue factor (Huang et al., 2016).
Endocrine pathways	G protein-coupled receptor	*Escherichia coli*	-It triggers the release of peptide hormones that directly stimulate the HPA axis via the vagus nerve (Herpich and Rincon, 2020).
	Tight junction protein	*Escherichia coli* and Bacteroides	The ionic permeability of the colonic epithelium is increased (Potter et al., 2022).
Immunization pathways	Aromatic hydrocarbon receptor	*Lactobacillus reuteri*	By triggering the IL-22 pathway, anti-inflammatory effects and intestinal barrier function are promoted (Qu et al., 2024).
	Lipopolysaccharide	Gram-negative bacteria of the *Enterobacteriaceae* family	Through the NF-κB activation pathway, it permeates systemic inflammation (Cuartero et al., 2024).
	Hp-CagA-IgG	*Helicobacter pylori*	It promotes the production of pro-inflammatory cytokines, cell adhesion molecules, and growth factors (Wang Y. et al., 2024).

### 3.3 Colony metabolite pathways

The gut microbiota produces various metabolites (Table 2), among them SCFAs are regarded as the most widely studied. SCFAs, primarily consisting of acetate, propionate, and butyrate, account for over 95% of the total metabolites (Li et al., 2019). These SCFAs are able to inhibit the nuclear factor-Kappa B (NF-κB) pathway and activate the Erk1/2 cascade response, thereby attenuating neuroinflammation and neuronal apoptosis after hippocampal injury. In addition, SCFAs can cross the BBB via monocarboxylate transporter proteins on endothelial cells and inhibit pathways associated with inflammatory responses (Chilton et al., 2024). It has been found that fecal SCFAs levels were significantly reduced in patients and experimental animals after occurrence of IS, but plasma SCFAs levels were decreased in experimental animals only (Parada Venegas et al., 2019). Human studies and animal experiments have shown that fecal SCFAs levels after IS exhibit a negative correlation with neurological scores, cerebral infarct volume and long-term functional prognostic scores in patients (Schneider et al., 2024; Chen et al., 2019). Bile acid is considered to be an efficient and multifunctional signaling molecule in digestive system (Thomas et al., 2008). It not only facilitates digestion of fats but also participates in regulating lipid, cholesterol, and blood sugar levels throughout the body by activating two important receptors (the FXR receptor in the cell nucleus and TGR5 receptor on the cell membrane), thereby helping to maintain homeostasis of immune system and energy (Collins et al., 2023). In the intestines, abnormal levels of bile acid have been found to culminate in excessive growth of bacteria in small intestine, which causes inflammation and damages intestinal epithelium. It is suggested that bile acids exert natural antibacterial activity through their “cleansing” capabilities via disruption of the cell membranes of bacteria (Xiao et al., 2021). The bile acids can activate the FXR pathway to promote expression of antimicrobial defense genes in the body, which aids protection of the gut against infection and damage, as well as prevention of pathogenic bacteria from entering blood circulation (Varesi et al., 2022). From these observations, it is evident that bile acids play a vital role in the maintenance of intestinal health. Besides bile acids, human body cannot synthesize certain amino acids on its own. Such amino acids, termed branched-chain amino acids (BCAAs, namely isoleucine, valine and leucine) are essentially absorbed from intestines and mainly obtained from diet (Zheng et al., 2023). The above-mentioned amino acids participate in several biochemical reactions and possibly impact the function of brain, thereby playing crucial role in nervous system. These amino acids are transported to the neurons across the BBB and are involved in transamination reactions (Chen C. et al., 2022). Glutamate and GABA synthesis are involved in transaminases of BCAAs in astrocytes with the amino group of glutamate being converted to glutamine. In contrast, changes in BCAA levels induced by SLC7A5 and branched-chain ketoacid dehydrogenase kinase may affect neuronal conductance, thereby accelerating alterations in cognitive function (Lemaitre et al., 2023). Certain gut microbiota, such as *Clostridium perfringens*, *E. coli*, and *Aspergillus* spp. produce Trimethylamine (TMA) with the participation of dietary nutrients (e.g., choline). Meanwhile, TMA is absorbed through the intestinal tract and subsequently transformed by monooxygenase in the liver to TMA N-oxide (TMAO) (Schneider et al., 2020). Following an IS, plasma TMAO levels in patients demonstrate dynamic fluctuations, initially rising and then falling during the acute phase, followed by a steady increase in the chronic phase (Parker et al., 2020). According to recent studies, TMAO can promote thrombosis in atherosclerosis by increasing the expression and activity of tissue factor, contributing to increased cardiovascular risk in IS patients. In addition, tryptophan acts as a precursor for kynurenine and other metabolites in microorganisms and in the host, which has anti-inflammatory properties in the GIT, and is thought to be neuroprotective (Cherian et al., 2019). After the onset of stroke, microorganisms that metabolize tryptophan, bile acids, BCAAs, and SCFAs may play an important role in the BBB and cerebral protection (Colombo et al., 2021). In contrast, TMAO-producing microbiota is metabolically active and may further exacerbate progression of stroke. Overall, changes in levels of colony metabolites can be monitored during stroke to improve the treatment strategy.

### 3.4 Endocrine pathways

The primary neuroendocrine stress response system is the hypothalamic–pituitary–adrenal (HPA) axis, which is involved in various mental activities, particularly crucial in emotional regulation (Młynarska et al., 2022). When the prefrontal cortex is inhibited and the amygdala is activated, both the sympathetic nervous axis and the HPA axis within the autonomic nervous system are simultaneously stimulated (Kumari et al., 2024). Among these, the sympathetic nervous system axis responds most rapidly, immediately releasing adrenaline to produce an immediate effect (Fan et al., 2024). Subsequently, the HPA axis is also activated as adrenaline secretion increases with hypothalamus paraventricular nucleus secreting corticotropin-releasing hormone (CRH), which subsequently activates synthesis and release of adrenocorticotropic hormone (ACTH) by the pituitary gland, ultimately leading to cortisol synthesis and release (Bai et al., 2022). Animal experiments have shown that in stroke model mice, those with reduced glucocorticoid receptor expression exhibited depressive-like behavior, while mice with increased glucocorticoid receptor expression demonstrated stronger stress resistance. Additionally, specialized sensory endocrine cells in the intestinal epithelium contain various G protein-coupled receptors, tight junction proteins, and other receptors related to fatty acids, carbohydrates, and amino acids (Yang et al., 2020). For example, metabolic products of *E. coli* can bind to these G protein-coupled receptors, thereby triggering the release of peptide hormones such as glucagon-like peptide-1 (GLP-1) (Chidambaram et al., 2022). These peptide hormones can act locally on intestinal neurons or activate neurons associated with the portal vein via the bloodstream, direct stimulation of cholinergic pathways and HPA axis through the vagus nerve, thereby inducing systemic anti-inflammatory responses (Zhou L. et al., 2022). Additionally, the metabolic products of *E. coli* and *B. anisopliae*, when bound to tight junction proteins could increase permeability of ion within colonic epithelium (Menke, 2019), thereby helping to reduce damage to tight junctions of intestines, lower permeability in intestines, and regulate alterations in the composition of microbial community (Zhang W. et al., 2023b; Kumar et al., 2021). In summary, gut microbiota and its metabolites exacerbate inflammatory responses and depressive symptoms after stroke by indirectly influencing the brain through impact on HPA axis but also directly acting on receptors of intestinal epithelia.

### 3.5 Immunomodulatory pathways

The gut is able to expeditiously sense irritants in the microenvironment of intestines, thereby serving as the immune barrier of the body (Xie et al., 2024). Based on the antigen presentation by dendritic cells, adaptive immune cells can differentiate in lymphoid-like structure of GIT into multiple effector subtypes (Zhang et al., 2019). Regulatory T cells, CD4+ effector cells (namely Th1, Th2, and Th17 cells) and cytotoxic CD8+ T cells are the effector subtypes that act locally and also migrate to other tissues, such as brain (Su et al., 2023). In particular, gut-derived IL17^+^ γδ T cells can promote post-stroke injury after migration to meninges (Wohleb et al., 2016). In addition, meninges-based γδ T cells act as a crucial factor for induction of anxious behavior by producing interleukin (IL)-17 (Schulthess et al., 2019). Tryptophan is metabolized into various metabolites such as indole-3-aldehyde by *Lactobacillus* species (including *Lactobacillus reuteri*, *L. acidophilus*, *L. murinus* and *L. johnsonii*) (Zelante et al., 2013). These indole metabolites activate the IL-22 pathway by binding to receptors of aromatic hydrocarbon (Bender et al., 2023). As a significant virulence factor of Gram-negative bacteria that belong to Enterobacteriaceae family, LPS mainly originates from the gut (Gomes et al., 2018) and can imbue systemic inflammation through activation of pathways like NF-κB and tumor necrosis factor-alpha (TNF-α) (Tang et al., 2021). Elevated levels of LPS in plasma of IS patients are associated with severe neurological deficits and poor stroke outcomes. Existing studies suggest that supplementation with probiotics can achieve reduced levels of LPS, alleviated neuroinflammatory responses and enhanced neurological function (Zhao et al., 2021; Zhang S. et al., 2024). Hp-CagA-IgG that is generated during chronic *Helicobacter pylori* (HP) infection triggers a systemic inflammatory response and alters certain blood components by increasing the release of growth factors, pro-inflammatory cytokines and cell adhesion molecules. Moreover, cerebral infarction and thrombosis can occur as a result of inflammation in areas with accumulated plaque (Margolis et al., 2021). Additionally, development and prognosis of IS is significantly influenced by intrinsic immunity activation (Simats et al., 2024). Excessive calcium ion overload in cytoplasm result in activation of microglia in the brain parenchyma during the hyperacute phase of IS, thereby culminating in release of increased levels of IL-1beta (IL-1β) and IL-18. Ischemic brain injury is markedly exacerbated by these pro-inflammatory mediators (Poh et al., 2019). In addition, numerous bloodstream-derived monocytes begin to infiltrate brain parenchyma within roughly 24 h of IS onset, which reached its peak after onset by the third day. In comparison with macrophages in the peripheral circulation, those that infiltrate brain tissue demonstrate distinguishable gene expression profiles. In particular, brain tissue-infiltrated macrophages exhibit upregulation of genes linked with phagocytic function and apoptotic cell recognition, namely principal regulatory genes in JAK/STAT pathway and TREM1 (Huang et al., 2016; Durán-Laforet et al., 2021). This phenomenon indicates specific functions in the regulation of local inflammatory responses and clearance of necrotic tissue. Neutrophils that migrate to the ischemic brain tissue and perivascular areas demonstrate functional impairments, including decreased phagocytic efficiency and increased generation of reactive oxygen species (ROS). These changes compound oxidative stress and lead to secondary injury in the brain tissue (Su et al., 2017). Gut microbiota imbalance is closely linked with the above-mentioned dysregulation of immune response. Based on earlier studies, scientists have suggested that inflammatory damage to brain tissue can be exacerbated by changes in the structure of gut microbiota, which result in disruption of immune homeostasis of the body and weakening of adaptive immune responses to post-stroke injury as well as promotion of pro-inflammatory factors release (Hermann et al., 2021). In addition, innate immune system can be activated by gut microbiota dysbiosis, which may be a significant mechanism that promotes the onset and progress of stroke. In particular, disruption of intestinal barrier integrity and subsequent translocation of microbial products are mechanism through which gut microbiota dysbiosis mainly activates the innate immune system (Buruş et al., 2021). This imbalance culminates in SCFAs depletion with their absence leading to increased intestinal permeability (leaky gut) since they are necessity for maintaining tight junctions of epithelial cells (Archana et al., 2024). As a classic member of pathogen-associated molecular pattern (PAMPs), LPS translocates into systemic circulation from the lumen of gut. Tissue-resident macrophages and circulating monocytes express pattern recognition receptors like toll-like receptor 4 (TLR4) that mainly recognize LPS (Akiba et al., 2020; Ropert, 2019). Downstream signaling cascades such as NF-κB pathway and NLRP3 inflammasome are triggered by binding of TLR4, which results in generation and release of pro-inflammatory mediators, namely interleukin-1β (IL-1β), IL-6 and TNF-α (Blevins et al., 2022). This cascade contributes to systemic inflammation by effectively shifting the innate immune system from a state of surveillance to a state of pro-inflammation. Notably, these immune alterations do not occur in isolation, but are closely interconnected with microbial metabolites, neuroactive signaling, and endocrine stress responses, together forming a complex regulatory network along the GBA.

### 3.6 Crosstalk among the four regulatory pathways

The four regulatory pathways discussed above do not act independently, but rather form an interconnected network that jointly shapes gut-brain communication after stroke (Hendel et al., 2026). Microbial metabolites appear to serve as an important hub within this network. For example, SCFAs can influence microglial homeostasis, thereby linking metabolic signaling with immune regulation in the CNS (Erny et al., 2015). In addition, microbiota-derived SCFAs may help preserve BBB integrity, suggesting a further link between microbial metabolism and neurovascular regulation (Fock and Parnova, 2023). In contrast, harmful microbial products such as TMAO and LPS are associated with vascular dysfunction, systemic inflammation, and aggravated neuroinflammatory responses (Dolkar et al., 2024).

Neurotransmitter signaling is also closely integrated with endocrine and immune responses. Increasing evidence indicates that the gut microbiota modulates metabolism and availability of neuroactive molecules such as 5-hydroxytryptamine, GABA, and glutamate, thereby influencing gut-brain signaling (Qu et al., 2024). In addition, selected microbial metabolites can regulate vagal afferent neuronal activity, thereby providing a neural route through which intestinal signals are transmitted to the brain (Jameson et al., 2025).

The endocrine and immune pathways likewise exhibit bidirectional regulation. After stroke, activation of the sympathetic nervous system and the hypothalamic–pituitary–adrenal axis alters intestinal permeability, motility, and microbial composition, which may in turn reshape gut-derived signaling (Xie et al., 2025). Conversely, pro-inflammatory cytokines and other immune mediators can further activate the hypothalamic–pituitary–adrenal axis, indicating that immune activation may provide feedback to neuroendocrine stress pathways (Konsman, 2023).

Importantly, intestinal barrier dysfunction and BBB impairment may serve as shared structural basis for this crosstalk. As these barriers are compromised, microbial products and inflammatory mediators can more readily enter the circulation and influence the brain, thereby amplifying interactions among these pathways (Aburto and Cryan, 2024). Therefore, gut-brain communication in stroke should be viewed as a dynamic and mutually regulated system rather than four isolated mechanisms.

## 4 Clinical application of MEAs in stroke

Dysphagia, sleep disorder, cognitive impairment, post-stroke depression, etc., are common stroke complications (Diener et al., 2022). Increased rates of disability and mortality as well as elevated risk of poor outcomes are markedly common in stroke patients due to the aforementioned complications (Benesch et al., 2021). Also, they drastically impair the quality of life, and impose a huge economic burden on families and society (Kumar et al., 2025). Relevant clinical studies have demonstrated that employing probiotics or other microbial modifiers can alter the composition of gut microbiota, thereby intervening in intestinal microecological balance (Tater and Pandey, 2021). This intervention seeks to lower the risk of stroke and increase prognosis of patient (Table 1). Generally, some bacterial species have been used as probiotic interventions for improving post-stroke outcomes. A clinical trial showed *Bifidobacterium longum* (OLP-01 strain) demonstrated improved cognitive function and altered gut microbiota of post-stroke patients, but the mechanism involve was not investigated (Lin et al., 2026).

### 4.1 Clinical application of MEAs in IS

The most common type of cerebrovascular disease is IS (Herpich and Rincon, 2020). Studies have shown that the frequency of opportunistic intestinal pathogens increases, while the number of commensal and beneficial bacteria decreases in IS patients (Amin et al., 2023; Potter et al., 2022). The traditional Chinese medicine formula ‘Charcoal Fire Decoction’ aims to promote growth of beneficial and commensal bacteria, competitively suppress opportunistic pathogens, thereby reducing sterile inflammation and platelet aggregation, and enhancing the host’s intestinal metabolism and immune function (Huang et al., 2024). A meta-analysis (Petrella et al., 2021) demonstrated that combination of probiotics with enteral nutrition following thrombolytic therapy provided multiple benefits for IS patients. This combined therapy significantly shortened hospital stay and bed rest duration, improved nutritional status, and increased levels of albumin, total serum protein, prealbumin, and hemoglobin. Although this therapy did not reduce the National Institutes of Health Stroke Scale score, it effectively alleviated the inflammatory response and significantly reduced levels of TNF-α, IL-6, and IL-10 (Wu and Chiou, 2021). Additionally, combination of probiotics and enteral nutrition further reduced the incidence of IS-related complications, lowered patient mortality, and decreased the risk of gut microbiota dysbiosis. Studies have also found a significant association between stroke and gut microbiota metabolites in patients. Specifically, three metabolites of dietary lipid phosphatidylcholine (choline, TMAO, and betaine) have demonstrated the ability to predict the risk of IS in a large clinical study (Li J. et al., 2024; Bonnechère et al., 2022). Electrical stimulation of vagus nerve has been widely applied to treat various diseases that affect the CNS, such as Parkinson’s disease, epilepsy, and depression (Lee et al., 2020). Recent works have demonstrated that this method can also ameliorate motor dysfunction in patients with stroke (Wang et al., 2021). However, the potential mechanism of vagus nerve electrical stimulation in stroke treatment may involve its regulatory effect on the gut microbiota, which requires further in-depth research. In summary, oral Chinese herbal formulations and probiotic supplements may enhance immunity and shorten treatment time in stroke patients.

The GBA is increasingly recognized as a therapeutic target in clinical management of IS. Post-stroke complications such as infections and neurological deficits can be potentially mitigated by MEAs, especially probiotics (Wei et al., 2023; Chen et al., 2025). The main focus of this therapeutic strategy is to counteract the post-stroke dysbiosis-induced metabolic deficits and barrier dysfunction by administering SCFA-producing strains to possibly restore homeostasis of gut microbiota and decrease inflammation (Tan et al., 2021).

The *Clostridium butyricum* strain MIYAIRI 588 has been found to potently produce butyrate in the human gut, and is regarded as safe and effective in protecting the neurons (Bertuccioli et al., 2023). Existing literature suggests that butyrate serves as main source of energy for colonocytes, upregulate proteins (e.g., occludin) of tight junction for intestinal barrier repair and systemic endotoxemia reduction. As a strong histone deacetylase inhibitor, butyrate promotes expression of anti-inflammatory regulatory T cells and inhibits NF-κB signaling in microglia under IS conditions (Hodgkinson et al., 2023). During preclinical studies, scientists observed that IS animal models that were supplemented with *C. butyricum* displayed reduced inflammatory biomarkers in serum and improved neurological deficit scores (Sun et al., 2016).

Production of lactate and acetate has been enhanced using multi-strain formulations combining *L. plantarum* and *B. longum* (Yang et al., 2024; Loo et al., 2025). Lowering of gut pH by these SCFAs can suppress overgrowth of pathogenic Enterobacteriaceae through intracellular acidification mediated by microbiota (Sorbara et al., 2019). The aforementioned examples clarify that function of MEAs as both dietary supplements and metabolic modulators that re-establish homeostasis of gut-brain, which offers a pragmatic adjunctive therapy for management of IS. Nevertheless, further research into these mechanisms will help us better prevent and treat stroke, while development of new treatment methods based on regulation the gut microbiota is urgently needed.

### 4.2 Clinical application of MEAs in hemorrhagic stroke

Hemorrhagic stroke is ranked as the second most prevalent cerebrovascular disease after IS (El Husseini et al., 2023). Clinically, secondary injuries such as perihematomal edema and neuroinflammation complicate management of hemorrhagic stroke, specifically intracerebral hemorrhage (ICH) (Sarma et al., 2019). Thus, current interventions for hemorrhagic stroke often target these secondary injuries, albeit limited effective treatment options. *Bifidobacterium tetragonum* tablets represent an innovative compounded tetragonum probiotic that, when administered orally, colonizes the intestines and effectively regulates the gut microbiota, promotes the growth of beneficial bacteria, improves intestinal biology, and enhances nutrient absorption (Fu et al., 2023). In that study, it was shown that administration of *B. bifidum* tetragonum tablets combined with an indwelling gastrostomy tube for enteral nutritional intervention after surgery for severe cerebral hemorrhage could improve intestinal mucosal barrier function, nutritional status, and gut microbiota of the organism, and reduce risk of complications (Cuartero et al., 2024). In addition, a study focusing on patients with hypertensive cerebral hemorrhage showed that compared with ordinary early enteral nutrition, gut microbiota imbalance and up-regulation of intestinal local immune function was beneficially improved by early enteral nutrition with synbiotics after surgery, thus reducing risk of complications. Meanwhile, a prospective study found that the gut microbiota of hypertensive cerebral hemorrhage patients differed significantly from that of healthy controls, as evidenced by decreased α-diversity, differences in β-diversity, increased abundance of potentially undesirable bacteria, decreased abundance of common SCFAs-producing bacteria, and decreased levels of fecal SCFAs (Mukhopadhya and Louis, 2025).

In preclinical models of ICH, *C. butyricum* has the potential to exhibit particular efficacy. Activation of NLRP3 inflammasome in microglia was suppressed by butyrate (Ganggang et al., 2025). This mechanism may accelerate resolution of hematoma and reduction of perihematomal edema thereby facilitating microglial polarization from M1 pro-inflammatory phenotype to M2 anti-inflammatory phenotype (Ziabska et al., 2026). Moreover, *C. butyricum* may restore barrier integrity of intestines, and minimize systemic translocation of LPS that would otherwise worsen cerebral edema (Liu et al., 2025).

As part of multi-strain formulations, *B. longum* may be used as probiotic intervention for hemorrhagic stroke. However, it seems most of these investigations have been mostly performed using clinical and preclinical models of IS. Thus, further studies are needed to mechanistically understand how acetate derived from specific strain of *B. longum* exhibits neuroprotective activities. These findings suggest that gut microbiota and its metabolite SCFAs could serve as regression markers and therapeutic targets for patients with hypertensive cerebral hemorrhage. Thus, prevention and treatment of hemorrhagic stroke may be effective through regulation of microbiota and concomitant enhancement of brain function.

### 4.3 Clinical application of MEAs in post-stroke recovery period

As evidenced in existing literature, gut microbiota is considered as an important factor in the post-stroke recovery and rehabilitation process (Wang Y. et al., 2024). In particular, increased scores of Mini-Mental State Examination (MMSE) and improved cognitive function were observed in IS patients after they had been supplemented with a beneficial bacterium called *Bifidobacterium* in a randomized double-blind controlled trial (Zhu et al., 2024). This finding indicates that gut microbiota regulation may serve as a novel approach for the promotion of post-stroke neurological recovery. In addition, scientists have discovered marked differences in metabolic parameters, including levels of serum triglycerides and biomarkers of insulin metabolism between healthy individuals and stroke patients (Sobngwi et al., 2012). Also, insulin metabolism and cognitive status in IS patients were improved following supplementation with combinations of bifidobacteria (such as *B. bifidum* and *L. fermentum*) and lactobacilli (such as *L. acidophilus* and *L. casei*) (Tamtaji et al., 2020). The abundance of Roseburia in the gut was substantially decreased, amid increased abundance of harmful pathogens in patients with stroke-associated pneumonia (SAP) (Xia et al., 2021). Thus, it has been suggested that early gut microbiota screening could provide crucial clues to predict SAP and assist in the development of corresponding treatment options since dysbiosis nearly doubled the risk of SAP (Ren et al., 2023). As evidenced by further studies, reduced number of SCFAs-producing bacteria has been observed in stroke-induced depression patients. Thus, gut health is significantly maintained by SCFAs, which may play a principal role in the promotion of recovery following stroke, thereby involving enhancing synaptic plasticity and facilitating cortical remodeling (Wang et al., 2022; Chen X. et al., 2022). Thus, rehabilitation outcomes and mental health status of stroke patients can be improved by combined therapy that incorporates SCFA supplementation, which has emerged as a new intervention for treatment of the condition. In a clinical trial, it was discovered that anxiety and depression symptoms could be alleviated in stroke patients that received formulated probiotics (containing *B. longum*, *L. acidophilus*, *L. fermentum*, *L. paracasei* and *L. rhamnosus*) via oral administration (Diener et al., 2022). In addition, early combination of *Bifidobacterium* triple capsules and enteral nutrition substantially ameliorated the nutritional status, immune function and neurological dysfunction of stroke patients with dysphagia (Johnson et al., 2023). Based on these findings, we can conclude that probiotics may potentially and therapeutically alleviate dysphagia, sleep disorder, cognitive impairment and post-stroke depression. Through regulation of gut microbiota and interaction with the brain (i.e., the GBA), probiotics could assist in improving clinical symptoms of patients and also impact on the pathological processes of the disease. Particularly, probiotics can regulate barrier function of intestine, suppress harmful bacteria proliferation, and promote beneficial bacteria growth, which reduces oxidative stress and systemic inflammatory responses, thus positively impacting the nervous system.

To address distinct pathophysiological challenges in IS, hemorrhagic stroke, and post-stroke recovery, the clinical application of MEAs across the two stroke types leverages bidirectional GBA. The primary therapeutic goal is restoration of intestinal barrier integrity in the acute phase of IS and hemorrhagic stroke (Shen et al., 2025; Wen et al., 2024). As stated in preceding sections, SCFA-producing strains like *C. butyricum*, could upregulate proteins of tight junction to protect integrity of intestinal barrier (Liu et al., 2020). In hemorrhagic stroke, this mechanism is significantly followed to mitigate perihematomal edema through inhibition of NLRP3 inflammasome activation, while systemic inflammation is ameliorated in IS by attenuating infarct expansion via suppression of microglial activation (Luo et al., 2019). There is a critical shift of focus to neuroplasticity and neuropsychiatric sequelae during the post-stroke recovery period (Aderinto et al., 2023). Post-stroke depression is associated with the neuroendocrine arm of GBA (especially the tryptophan-serotonin pathway), which is disrupted by persistent dysbiosis. Thus, MEAs can promote synaptic remodeling and improve cognitive and emotional outcomes by regulating metabolism of tryptophan and enhancing brain-derived neurotrophic factor via restoration of microbial homeostasis (Zhou et al., 2023; Zhou B. et al., 2022; Li L. et al., 2024). It is possible that MEAs would offer a holistic therapeutic strategy that transitions from acute neuroprotection through restoration of intestinal barrier to chronic rehabilitation via modulation of neuroendocrine arm of GBA. Nonetheless, there is the need to conduct further research to understand how probiotics exert their beneficial effects via regulation of gut microbiota and GBA. The significance of gut microbiota in the rehabilitation of stroke has been highlighted in current studies, thereby providing a conceptual foundation to develop novel gut microbiota-based treatment strategies. With the aim of generating new insights that support development of personalized medical approaches, future studies should investigate specific ways in which various probiotic strains modulate gut microbiota composition and affect host health.

## 5 Conclusion

Stroke has become an increasingly significant burden on public health as the global population continues to age. The critical role of gut microbiota in the onset, progression, and prognosis of stroke is revealed by the concept of GBA. This article summarizes existing research and proposes that the body primarily achieves brain-gut communication through four main pathways, namely the neurotransmitter, the microbial metabolite, the endocrine, and immune regulatory pathways. Further classification and summarization of the effects of bacterial microbiota on these four pathways and their regulatory mechanisms related to stroke are provided. As a potential intervention pathway and therapeutic target for neurological disorders, gut microbiota has gained much attention in recent times. Clinical applications targeting gut microbiota include oral probiotic formulations, electroacupuncture stimulation of the vagus nerve, and methods targeting immune or endocrine regulation. Also, we discovered improved nutritional status and neurological dysfunction in stroke patients by some probiotic formulations. Despite their considerable potential, the clinical use of microbial ecological interventions in neurological disorders is still at an early stage, hence their safety and therapeutic effectiveness should be established via further rigorous studies. Utilization of multi-omics technologies and large-scale, high-quality, multi-center clinical trials should be the focus of future research to develop microbial ecological preparations with clear mechanisms of action and stable efficacy. In summary, a deep understanding of the complex interactions between the gut microbiota and the GBA is crucial for the development of novel and effective stroke treatments. By integrating modern biotechnology and clinical research methods, it is hoped that more potential therapeutic targets can be identified, thereby improving stroke patients’ quality of life.
